# Zhenbao pill protects against acute spinal cord injury via *miR-146a-5p* regulating the expression of GPR17

**DOI:** 10.1042/BSR20171132

**Published:** 2018-01-19

**Authors:** Yongxiong He, Bokang Lv, Yanqiang Huan, Bin Liu, Yutang Li, Lizhou Jia, Chenhui Qu, Dongsheng Wang, Hai Yu, Hongwei Yuan

**Affiliations:** 1 Department of Spine Surgery, Inner Mongolia People’s Hospital, Hohhot 010017, Inner Mongolia, People’s Republic of China.; 2 Department of Clinic, Inner Mongolia Medical University People’s Hospital, Hohhot 010020, Inner Mongolia, People’s Republic of China.; 3 Department of Pathology, Inner Mongolia Medical University, Hohhot 010050, Inner Mongolia, People’s Republic of China.

**Keywords:** ASCI, cell apoptosis, GPR17, miR-146a-5p, zhenbao pill

## Abstract

The aim of the present study was to observe the effect of zhenbao pill on the motor function of acute spinal cord injury (ASCI) rats and the molecular mechanisms involving *miR-146a-5p* and G-protein-coupled receptor 17 (GPR17). ASCI rat model was established by modified Allen method, and then the rats were divided into three groups. SH-SY5Y cells were cultured overnight in hypoxia condition and transfected with *miR-146a-5p* mimic or *miR-146a-5p* inhibitor. The hind limb motor function of the rats was evaluated by Basso, Beattie, Bresnahan (BBB) scoring system. Quantitative real-time PCR (qRT-PCR) and Western blot were used to detect the expression of *miR-146a-5p*, GPR17, inducible nitric oxide synthase (iNOS), interleukin 1β (IL-1β), and tumor necrosis factor α (TNF-α). Neuronal apoptosis was measured using flow cytometry assay. Luciferase reporter assay was performed to determine the regulation of *miR-146a-5p* on GPR17. Zhenbao pill could enhance hind limb motor function and attenuate the inflammatory response caused by ASCI. Moreover, zhenbao pill increased the level of *miR-146a-5p* and decreased GPR17 expression *in vivo and in vitro*. Bioinformatics software predicted that GPR17 3′-UTR had a binding site with *miR-146a-5p*. Luciferase reporter assay showed that *miR-146a-5p* had a negative regulatory effect on GPR17 expression. Knockdown of *miR-146a-5p* could reverse the effect of zhenbao pill on the up-regulation of GPR17 induced by hypoxia, reversed the inhibitory effect of zhenbao pill on the cell apoptosis induced by hypoxia and the recovery of zhenbao pill on hind limb motor function in ASCI rats. Zhenbao pill could inhibit neuronal apoptosis by regulating *miR-146a-5p*/GPR17 expression, and then promoting the recovery of spinal cord function.

## Introduction

Acute spinal cord injury (ASCI) is a high disability of the central nervous system injury caused by trauma. ASCI is mainly caused by various accidents, sports, natural disasters, falls, and violence, and its incidence has been high. According to statistics, the incidence of ASCI in developed countries is 236–1009 per million people [1]. In China, annual incidence (6.7 cases per million people) is lower than the developed countries. Therefore, it is of great significance to strengthen the study of spinal cord injury (SCI). SCI can be divided into primary injury and secondary injury [2]. Primary injury mainly refers to the destruction of the spinal cord structure caused by external forces. Secondary injury is based on the primary injury, and is continuous damage caused by the inflammatory response, immune injury, and cell apoptosis on the injured spinal cord tissue, which is the main cause of spinal cord dysfunction [3,4]. Primary damage to the spinal cord function and structure is irreversible. Therefore, the study of ASCI treatment and rehabilitation is focussed on secondary injury [5].

Zhenbao pill is composed of 29 traditional Chinese medicines, including pearl, cassia tora, bezoar, saffron, amomum tsao-ko, and licorice etc. It can promote blood circulation, activate collaterals, and soothe the nerves. In clinical usage, zhenbao pill is used for the treatment of nervous system diseases, such as stroke and hemiplegia sequelae. Modern pharmacological studies have shown that zhenbao pill has the effect of repairing damaged nerve cells, promoting microcirculation, and scavenging oxygen free radicals [6]. Studies have shown that zhenbao pill has a protective effect on nerve cell injury in brain tissue and cerebral edema caused by incomplete cerebral ischemia/reperfusion. It can reduce red cell assembling index, platelet aggregation rate and whole blood viscosity, improve cerebral blood flow (CBF), expand micrangium and enhance body’s antioxidant capacity [7]. Wang et al. [8] found that zhenbao pills could significantly improve the neurological function after cerebral ischemia/reperfusion in rats. Hashengaowa [9] reported that zhenbao pills had a good therapeutic effect on neurotoxicity induced by vincristine. Additionally, Yu et al. [10] found that zhenbao pills had certain protective effects on nerve in rats with SCI. However, the basic study on zhenbao pill in the treatment of SCI has been poorly reported.

MiRNA is a class of noncoding small RNAs with a length of ~20 nts. MiRNAs can combine with RNA-induced silencing complex (RISC) and complement with target mRNA, and then affect the expression of target genes [11]. In recent years, miRNA has been widely involved in the regulation of various pathophysiological processes such as inflammation, growth and development, cell apoptosis, and tumor [12,13]. Several studies have reported that a large number of miRNAs are de-regulated after ASCI [14,15], suggesting that miRNAs may be involved in the regulation of ASCI. The results of bioinformatics analysis of miRNAs whose expression levels have changed after ASCI suggest that these miRNAs are actively involved in the pathogenesis of ASCI in rats. They play a role in a variety of mechanisms such as cell apoptosis, oxidative stress, angiogenesis, and inflammatory response, suggesting that the change in these miRNAs’ expression may be related to the occurrence and development of secondary SCI. In our study, we found abnormal expressions of five miRNAs in ASCI rats, and only *miR-146a-5p* expression was increased after being treated with zhenbao pill. Additionally, *miR-146a-5p* has been reported to relieve neuropathic pain in spinal cord [16,17]. Together, we speculated that *miR-146a-5p* might be regulated to the occurrence and development of ASCI. Thus, *miR-146a-5p* was investigated in the present study.

G-protein-coupled receptor 17 (GPR17) is an orphan receptor that can be double-activated by uracil and cysteinyl-leukotriene (CysLT) receptor families [18]. GPR17 is mainly distributed in the brain, kidney, and heart, and in the brain, GPR17 is mainly distributed in white matter, gray matter, subependymal zone, and corpus callosum. At present, some studies have shown that GPR17 plays an important role in SCI, myelin sheath injury, cerebral ischemic injury, and the regulation of oligodendrocytes and neuronal differentiation [19]. In the mice model of SCI, GPR17 mainly mediates neuronal death in the early stage of injury, and in the advanced stage of injury, it mediates the migration of microglia/macrophages to the injured area. The tissue damage after GPR17 knockdown is significantly reduced [20], suggesting that GPR17 is a potential therapeutic target for brain injury and SCI. Moreover, bioinformatics analysis found that GPR17 3′-UTR had a binding site with *miR-146a-5p*. Therefore, it is of great significance to explore the role of GPR17 in related diseases and to screen the related therapeutic drugs.

In the present study, we investigated the effect of zhenbao pill on the motor function of ASCI rats and the molecular mechanisms involving *miR-146a-5p* and GPR17, aiming to find new therapeutic targets for ASCI.

## Materials and methods

### Animals

Forty-nine young adult female SD rats (180–220 g) were included in the study. Twenty-one of them were randomly divided into three groups: sham operation group (sham group, *n*=7), ASCI group (*n*=7), and zhenbao pill treatment group (ASCI + zhenbao pill group, *n*=7). Others (*n*=28) were randomly assigned into four groups: sham group (*n=*7), ASCI group (*n*=7), ASCI + zhenbao pill + negative control (NC) group (*n*=7), and ASCI + zhenbao pill + *miR-146a-5p* inhibitor group (*n*=7). Rats in ASCI + zhenbao pill + *miR-146a-5p* inhibitor group received *miR-146a-5p* inhibitor vector packaged with lentiviral vector by intraperitoneal injection. It is worth mentioning that whenever miR inhibitor was used, the control group received a NC with the same vehicle. The Care and Use of Laboratory Animals issued by the Chinese Association for Laboratory Animal Care approved all procedures.

### ASCI model

In the present study, ASCI model was established by modified Allen method. Rats were anesthetized by intraperitoneal injection of 3% pentobarbital before operation. Then, rats received a laminectomy at T10 vertebra after a dorsal skin incision. The spinal cord was contused using an impactor (2.4 mm diameter, 10 g weight) from the height of 2.5 cm along the guide needle vertical strike T10. Rats in sham group were treated similarly, but without being contused. Postoperative care included once or twice daily artificial micturition and defecation. For ASCI + zhenbao pill group, 0.6 g/(kg.day) zhenbao pill was administrated by intragastric administration for 2 weeks. ASCI group and sham group were given the same dose of saline.

### Locomotor assessment

The hind limb motor function of the rats was evaluated by the Basso, Beattie, Bresnahan (BBB) scoring system [21]. The BBB scoring criteria was divided into 21 scores. Normal rats have a BBB score of 21, while ASCI rats with completely paralyzed hind limbs have a BBB score of 0. The hind limb motor function of the rats at 1st, 3rd, 7th, 14th, and 21st day after ASCI were tested.

### Spinal cord samples

At different time points (12, 24, and 72h), the rats of each group (sham group, ASCI group, and ASCI + zhenbao pill group) were killed. The spinal cord tissues were obtained and stored at −80°C for further study.

### Flow cytometry assay

The spinal cord tissue was taken from ASCI rats and digested with 2.5 g/l trypsin for 10 min, and then filtered through a 200-mesh sieve. Next, centrifugation was done at 1000 rpm for 5 min and the supernatant was discarded. Then, propidium iodide solution was added for 30 min. The cells’ apoptosis rate was detected by flow cytometry (Becton Dickinson, U.S.A.).

### Quantitative real-time PCR

According to the directions of reagent, the total RNA of spinal cord (4 mm length; 2 mm from the injured epicenter cranially and caudally, respectively) and SH-SY5Y cells were extracted by TRIzol reagent (Invitrogen, Carlsbad, CA). The Taqman miRNA Reverse Transcription Kit (Applied Biosystems) was used to perform reverse transcription of miRNA according to manufacturer’s instructions. PCR amplification of miRNA was performed on a fluorescence quantitative PCR instrument. The reaction conditions of PCR amplification were performed at 95°C for 30 s, followed by 40 cycles of thermal cycling at 95°C for 5 s, and 60°C for 30 s. And according to the manufacturer’s instructions, the standard SYBR Green RT-PCR Kit (Applied Biosystems) was used to detect the mRNA level of GPR17. The expressions of *miR-146a-p*, GPR17, inducible nitric oxide synthase (iNOS), tumor necrosis factor α (TNF-α), and interleukin 1β (IL-1β) were analyzed by 2^−ΔΔ*C*^_t_ method and GAPDH was used as a control for normalization. The following primers were used: *miR-146a-5p* forward, 5′-TGA GAA CTG AAT TCC ATG GGT-3′; iNOS forward, 5′-GAA AGA GGA ACA ACT ACT GCT GGT-3′ and reverse, 5′-GAA CTG AGG GTA CAT GCT GGA GC-3′; IL-1β forward, 5′-GAG AGA CAA GCA ACG ACA AAA TCC-3′ and reverse, 5′-TTC CCA TCT TCT TCT TTG GGT ATT-3′; TNF-α forward, 5′-CTT CTG TCT ACT GAA CTT CGG GGT-3′ and reverse, 5′-TGG AAC TGA TGA GAG GGA GCC-3′; and GAPDH forward, 5′-TGT TCC TAC CCC CAA TGT G-3′ and reverse, 5′-GTG TAG CCC AAG ATG CCC T-3′.

### Western blot

According to the manufacturer’s specifications, total protein was extracted from tissues or cells using RIPA lysis buffer (Bolingkewei, Beijing) and protein concentration of each sample was determined by BCA kit (Bolingkewei, Beijing). The protein was separated by SDS/PAGE and then transferred on to PVDF membranes (Millipore, U.S.A.). The membranes were blocked with 5% skim milk and incubated with anti-GPR17 antibody (1:1000, Abcam, U.K.) and anti-β-actin antibody (1:5000, Abcam, U.K.) overnight at 4°C. The membranes were then incubated with the goat anti-mouse secondary antibody for 2 h. The proteins were visualized by ECL chemiluminescence method.

### Luciferase reporter assay

The binding sites of *miR-146a-5p* and GPR17 were analyzed online using TargetScan [22] and microRNA.org. (http://www.microrna.org/microrna/getGeneForm.do). According to the complementary binding sites predicted by software, PCR amplification primers were designed and the GPR17 3′-UTR fragment (610 bp) containing *miR-146a-5p* sequence was constructed. Then, GPR17 3′-UTR fragment was cloned into the PGL3 dual-luciferase reporter vector (Promega, WI, U.S.A.), and named as GPR17-3′-UTR-WT. On the other hand, a single target site mutant GPR17-3′-UTR-MUT was constructed by using PCR technique for site-directed mutagenesis. SH-SY5Y cells were co-transfected with GPR17-3′-UTR-WT reporter vector or GPR17-3′-UTR-MUT reporter vector and *miR-146a-5p* mimic or *miR-146a-5p* inhibitor or NC by using Lipofectamine 2000 (Invitrogen). The cells were lysed after transfection for 24 h, and the luciferase activity was detected by double luciferase reporter (DLR) assay system following the manufacturer's instructions.

### Cell cultures

SY-SH-5Y cells were cultured in DMEM/F12 medium (Solarbio, U.S.A.) containing 10% FBS, 100 mg/l penicillin and 100 mg/l streptomycin, and incubated in an incubator (37°C, 5% CO_2_). The cells used in the experiment were in the logarithmic growth phase.

### Cell transfection

SH-SY5Y cells were cultured overnight at 37°C with 5% CO_2_, 1% O_2_, and 94% N_2_. *MiR-146a-5p* mimic or *miR-146a-5p* inhibitor were transfected into the cells using Lipofectamine 2000 (Invitrogen) following the manufacturer’s instructions. Then, the cells were collected for related detection after 48 h of transfection.

### Statistical analysis

Data were analyzed by SPSS 20.0 software, and all measurement data were expressed as mean ± S.D. Student’s *t* test was used to calculate the statistical differences between two groups. Comparisons amongst multiple groups were analyzed using a one-way ANOVA. *P*<0.05 was regarded as statistically significant.

## Results

### Zhenbao pill attenuated the inflammatory response caused by ASCI

**Figure 1:**
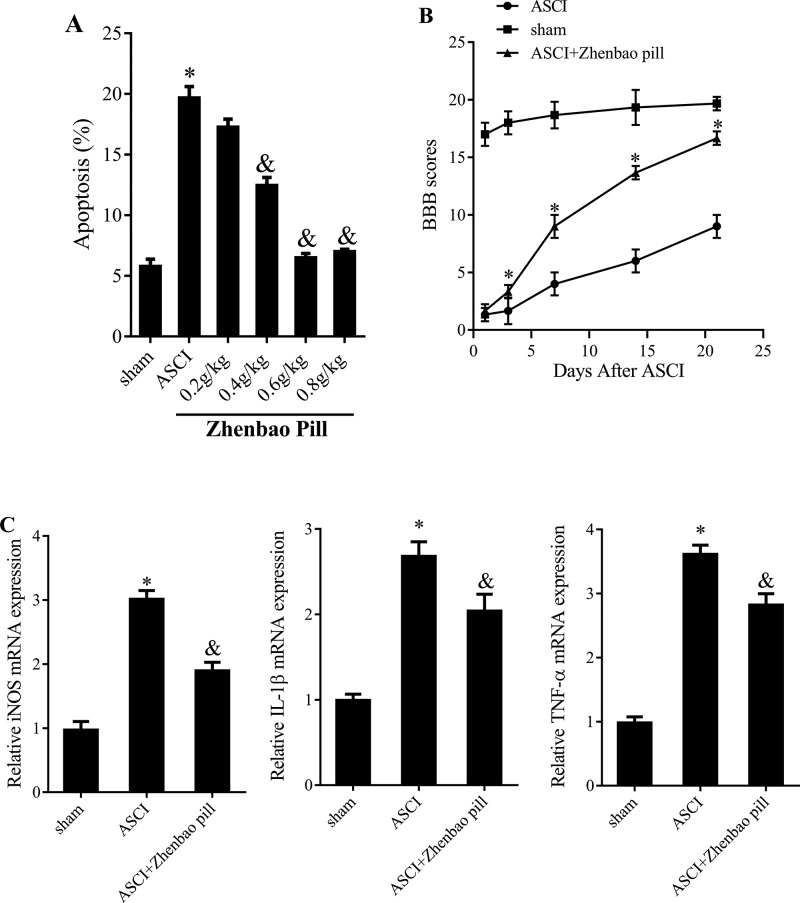
Effect of zhenbao pill on hind limb motor function and the expression of iNOS, TNF-α, and IL-1β Rats were divided into sham group, ASCI group, and zhenbao pill treatment group (*n*=7). ASCI model was established by modified Allen method. (**A**) Flow cytometry assay was used to measure neurones’ apoptosis. (**B**) BBB scoring system was used to evaluate hind limb motor function of these rats. Normal rats have a BBB score of 21, while ASCI rats with completely paralyzed hind limbs have a BBB score of 0. (**C**) Quantitative real-time PCR (qRT-PCR) was used to detect the mRNA expressions of iNOS, IL-1β and TNF-α. *P<0.05, compared with sham; ^&^*P*<0.05, compared with ASCI.

In order to choose the optimum concentration, we carried out zhenbao pill concentration gradient tests. The study selected four concentrations (0.2, 0.4, 0.6, 0.8 g/kg) for pre-experiments. Results showed that the inhibitory effect of zhenbao pill on neurones’ apoptosis was not obvious at 0.2 g/kg dosing conditions, but the inhibitory effect increased with increasing dosage (Figure 1A). The inhibitory effect of zhenbao pill on neurones’ apoptosis was strongest at the concentration of 0.6 g/kg (Figure 1A). Thus, 0.6 g/kg was chosen as the therapeutic dose for further study. In animal experiments, rats were divided into three groups (*n*=7/group): sham operation group, ASCI group, zhenbao pill treatment group. The hind limb motor function of these rats was evaluated by the BBB scoring system. Normal rats have a BBB score of 21, while ASCI rats with completely paralyzed hind limbs have a BBB score of 0. We test the hind limb motor function at 1st, 3rd, 7th, 14th, and 21st day after ASCI. With the prolongation of time, BBB score of ASCI group and ASCI + zhenbao pill group increased gradually (Figure 1B). However, at each time point, the BBB scores of ASCI + zhenbao pill group were markedly higher than ASCI group (Figure 1B). Moreover, the expressions of iNOS, IL-1β, and TNF-α in spinal cord of ASCI rats were significantly increased compared with sham operation group, while the expressions were highly attenuated in zhenbao pill treatment group (Figure 1C). These results indicated that zhenbao pill could markedly attenuate the inflammatory response caused by ASCI.

### Zhenbao pill up-regulated *miR-146a-5p*

**Figure 2:**
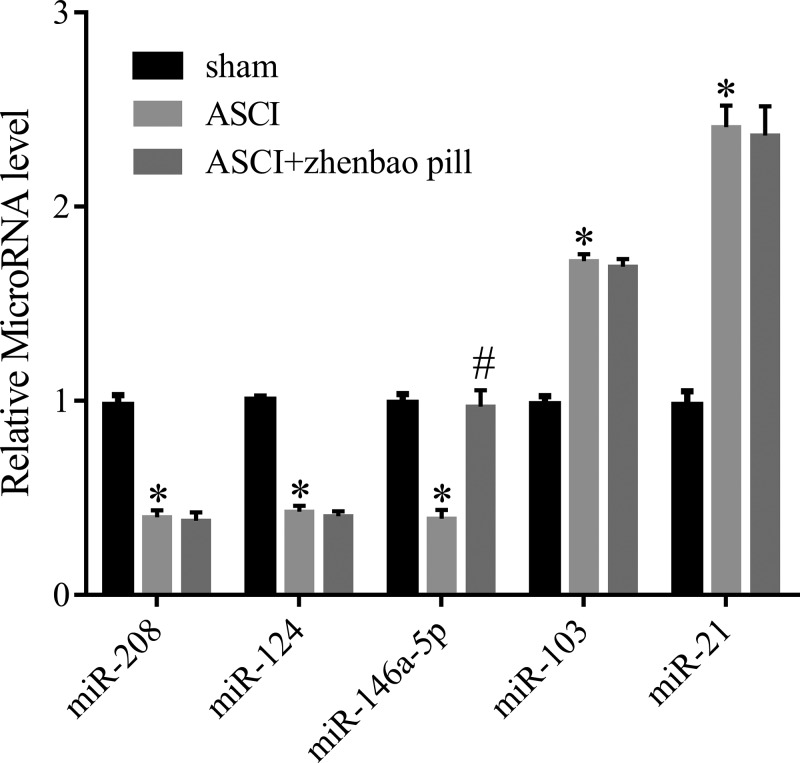
The expression of miRNA in ASCI rats Quantitative real-time PCR (qRT-PCR) was used to detect miRNA expression in ASCI rats. In the ASCI group, the levels of *miR-208, miR-124*, and *miR-146a-5p* were decreased, while the levels of *miR-103* and *miR-21* were increased. In the ASCI + zhenbao pill group, *miR-146a-5p* expression was increased, the levels of other miRNA had no significant change. *P<0.05, compared with sham; ^#^*P*<0.05, compared with ASCI.

In this part, we selected five miRNAs to test according to the literature [16,23–28], aiming to confirm which miRNA could be affected by zhenbao pill grouping method described above. The results showed that *miR-208, miR-124, miR-146a-5p, miR-103*, and *miR-21* were all expressed abnormally in spinal tissue of ASCI rats (Figure 2). However, only *miR-146a-5p* expression was increased after being treated with zhenbao pill (Figure 2). These results indicated that zhenbao pill might exert its effects on ASCI by regulating *miR-146a-5p*.

### Zhenbao pill regulated *miR-146a-5p* and GPR17 expression and neuronal apoptosis

**Figure 3:**
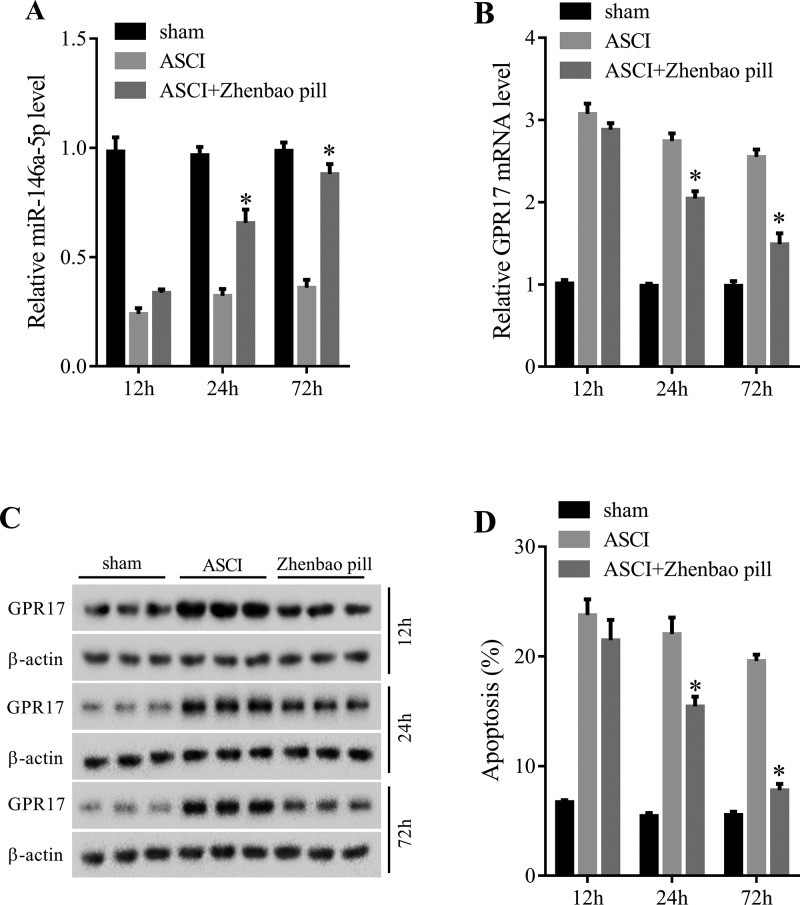
Effect of zhenbao pill on *miR-146a-5p* and GPR17 expression and neuronal apoptosis (**A**) The levels of *miR-146a-5p* in spinal tissue of rats at different time points. (**B**) The mRNA expressions of GPR17 in spinal tissue of rats at different time points. (**C**) The protein levels of GPR17 in spinal tissue of rats at different time points. (**D**) Flow cytometry assay was used to measure the effect of zhenbao pill on neuronal apoptosis. *P<0.05, compared with ASCI.

As mentioned above, GPR17 might be a potential therapeutic target for SCI and *miR-146a-5p* might be associated with ASCI, so we investigated the role of zhenbao pill on the expression of *miR-146a-5p* and GPR17. It could be seen that *miR-146a-5p* expression was decreased (Figure 3A) and GPR17 expression was increased (Figure 3B,C) after ASCI, but zhenbao pill could reverse these, and the effects of zhenbao pill on the expression of *miR-146a-5p* and GPR17 were increased as time goes on (Figure 3A,B). Flow cytometry assay was used to measure the effect of zhenbao pill on neuronal apoptosis. The result showed that neuronal apoptosis was increased after ASCI. While zhenbao pill could inhibit neuronal apoptosis, the inhibitory effect showed an increasing trend over time (Figure 3D).

### *MiR-146a-5p* directly regulates GPR17 expression

**Figure 4:**
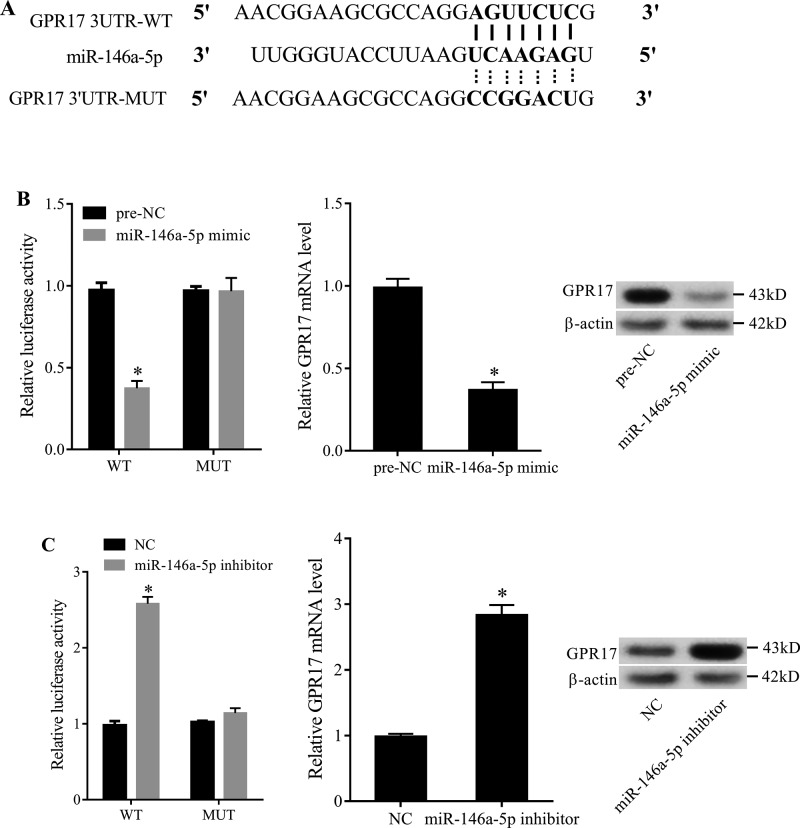
The effect of *miR-146a-5p* on GPR17 expression (**A**) The results predicted by online bioinformatics methods showed that GPR17 3′-UTR contained an *miR-146a-5p* binding site. The solid line indicated that *miR-146a-5p* and GPR17 could be combined, and the dotted line indicated that the binding site was mutated. (**B**) The luciferase activity of GPR17 3′-UTR and the expression of GPR17 in SH-SY5Y cells co-transfected with *miR-146a-5p* mimics and LRP. (**C**) The luciferase activity of GPR17 3′-UTR and the expression of GPR17 in SH-SY5Y cells co-transfected with *miR-146a-5p* inhibitor and LRP. *P<0.05, compared with pre-NC or NC (pre-NC was the control group of *miR-146a-5p* mimic. NC was the control group of *miR-146a-5p* inhibitor).

Based on bioinformatics analysis (TargetScan and microrna.org), we found that GPR17 3′-UTR had a binding site with *miR-146a-5p* (Figure 4A). Then, we used DLR assay to detect the regulatory relationship between *miR-146a-5p* and GPR17. The luciferase reporter plasmid (LRP) containing wild-type (WT) gene of GPR17 3′-UTR or mutant (MUT) gene of GPR17 3′-UTR was constructed, and SH-SY5Y cells were co-transfected with *miR-146a-5p* mimics and LRP. DLR assay showed that the luciferase activity of WT GPR17 3′-UTR in SH-SY5Y cells co-transfected with *miR-146a-5p* mimics was decreased, while the luciferase activity of MUT GPR17 3′-UTR had no signficant change (Figure 4B). Moreover, *miR-146a-5p* mimics reduced the mRNA and protein expression of GPR17 in SH-SY5Y cells (Figure 4B). Compared with *miR-146a-5p* mimics, *miR-146a-5p* inhibitor had an opposite trend (Figure 4C).

### Zhenbao pill regulated the expression of *miR-146a-5p* and GPR17 in SH-SY5Y cells exposed to hypoxia

**Figure 5:**
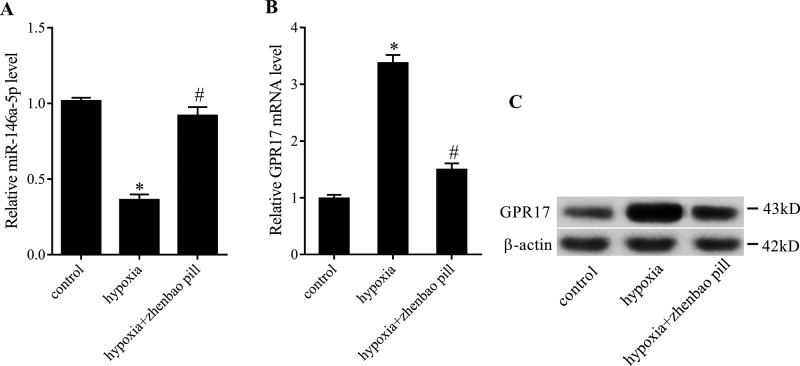
The effect of zhenbao pill on the expression of *miR-146a-5p* and GPR17 in SH-SY5Y cells exposed to hypoxia. SH-SY5Y cells were divided into three groups: control group, hypoxia group, and pretreatment with zhenbao pill group. (**A**) The level of *miR-146a-5p* in cells was measured by qRT-PCR. (**B**) The mRNA and protein level of GPR17 in cells were examined by qRT-PCR and Western blot. *P<0.05, compared with control; ^#^*P*<0.05, compared with hypoxia.

Because of its similar characteristics to nerve cells, SH-SY5Y human neuroblastoma cells are commonly used as a substitute for nerve cells [29]. In this study, SH-SY5Y cells were divided into three groups: control group, hypoxia group (cells were cultured overnight at 37°C with 5% CO_2_, 1% O_2_ and 94% N_2_), and pretreatment with zhenbao pill group. Hypoxia stimulation is a method of simulating damage. The expression of *miR-146a-5p* in hypoxia group was reduced as measured by quantitative real-time PCR (qRT-PCR), and zhenbao pill pretreatment reversed the expression of *miR-146a-5p* (Figure 5A). In addition, the mRNA and protein level of GPR17 in SH-SY5Y cells were increased in hypoxia group, as expected, zhenbao pill treatment decreased the expression of GPR17 (Figure 5B).

### *MiR-146a-5p* reversed the role of zhenbao pill on GPR17 expression

**Figure 6:**
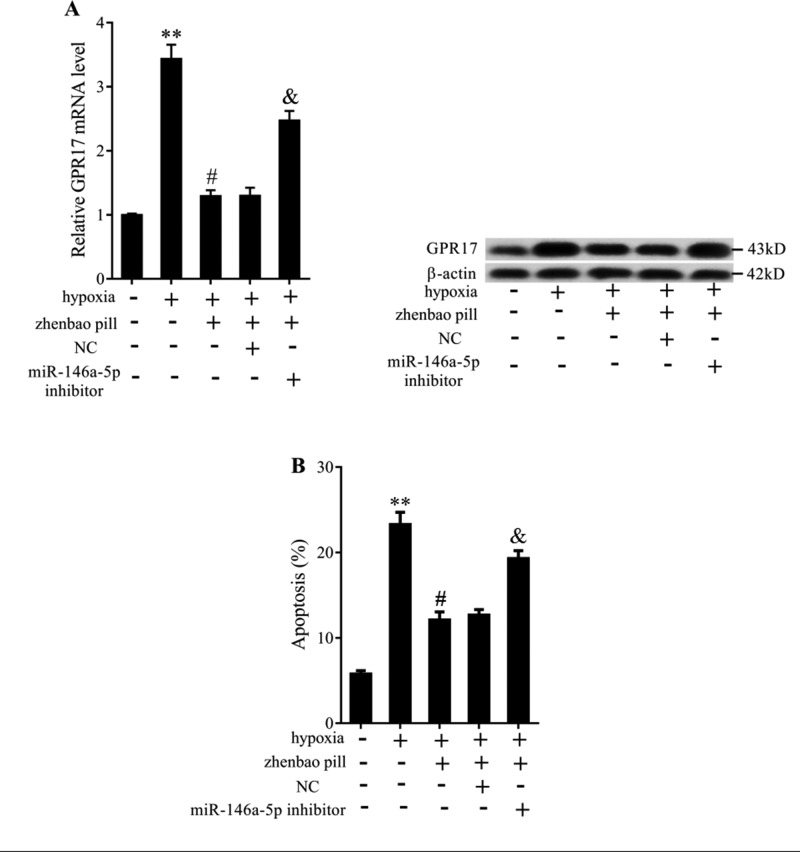
The effect of *miR-146a-5p* on the expression of GPR17 induced by hypoxia. (**A**) The mRNA and protein level of GPR17 in SH-SY5Y cells exposed to hypoxia or pretreated with zhenbao pill or transfected with *miR-146a-5p* inhibitor. (**B**) Flow cytometry assay was used to measure the effect of *miR-146a-5p* inhibitor on SH-SY5Y cells’ apoptosis. **P<0.01, compared with control; ^#^*P*<0.05, compared with hypoxia; ^&^*P*<0.05, compared with hypoxia + zhenbao pills + NC. NC was a negative control for inhibitor.

To explore the regulatory relationship between *miR-146a-5p* and zhenbao pill on GPR17 expression, SH-SY5Y cells were divided into five groups: control group, hypoxia group, hypoxia + zhenbao pill group, hypoxia + zhenbao pill + NC group, and hypoxia + zhenbao pill + *miR-146a-5p* inhibitor group. As shown in Figure 6A, zhenbao pill reduced the expression of GPR17, while knockdown of *miR-146a-5p* reversed the down-regulation of GPR17. Additionally, flow cytometry assay showed that the apoptosis of SH-SY5Y cells exposed to hypoxia was increased, and zhenbao pill inhibited cell apoptosis. However, knockdown of *miR-146a-5p* could reverse the role of zhenbao pill in SH-SY5Y cells’ apoptosis (Figure 6B). The results indicated that zhenbao pill could reduce cell apoptosis via down-regulating GPR17, while this could be reversed by *miR-146a-5p* inhibitor.

### Zhenbao pill promotes the recovery of spinal cord function via regulating *miR-146a-5p*/GPR17

**Figure 7:**
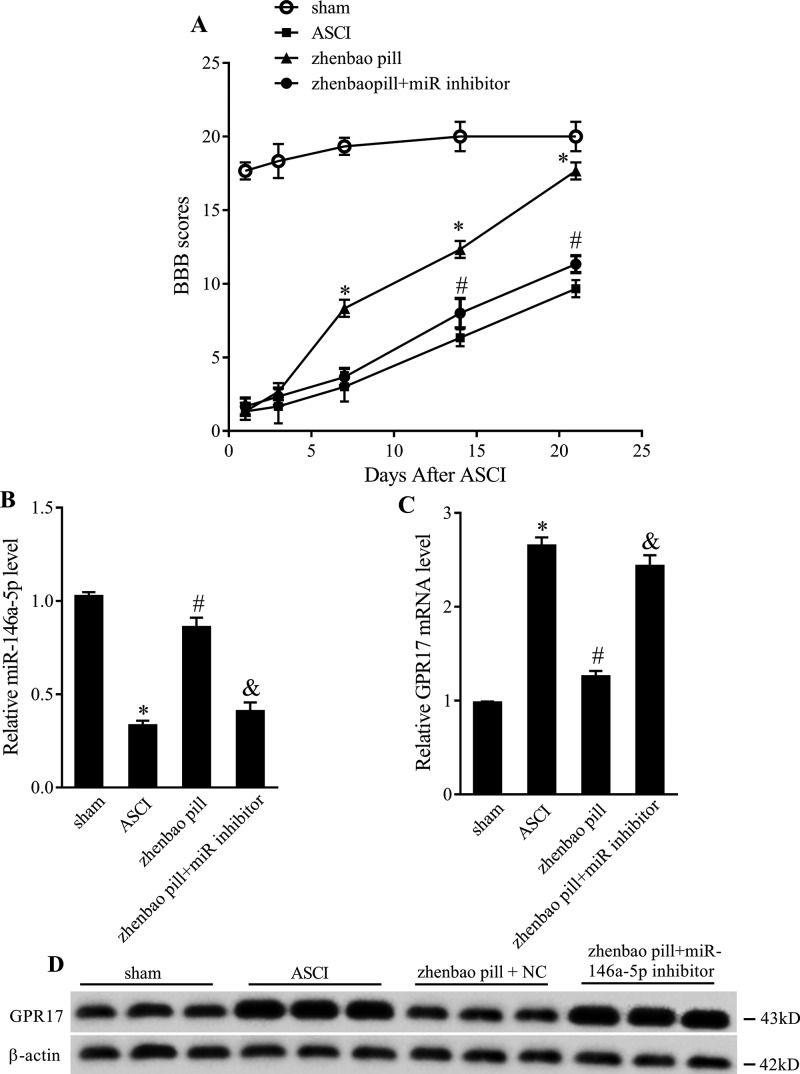
Effect of *miR-146a-5p* on hind limb motor function. The rats were divided into four groups: sham group, ASCI group, ASCI + zhenbao pill + NC group, and ASCI + zhenbao pill + *miR-146a-5p* inhibitor group. (**A**) BBB scoring system was used to evaluate the hind limb motor function. (**B**) The expression of *miR-146a-5p*. (**C**) The mRNA expression of GPR17. (**D**) The protein expression of GPR17. *P<0.01, compared with sham; ^#^*P*<0.05, compared with ASCI; ^&^*P*<0.05, compared with ASCI + zhenbao pill + NC. NC was a negative control for inhibitor.

In the part, we verified the mechanism of zhenbao pill on ASCI *in vivo*. Twenty-eight adult female SD rats were divided into four groups: sham group, ASCI group, ASCI + zhenbao pill + NC group, and ASCI + zhenbao pill + *miR-146a-5p* inhibitor group. BBB scoring system was used to evaluate the hind limb motor function at 1st, 3rd, 7th, 14th, and 21st day after ASCI. The sham and the ASCI groups were the same as in Figure 1B. The ASCI + zhenbao pill + NC group was also the same of the ASCI + zhenbao pill in Figure 1B. As shown in Figure 7A, zhenbao pill could promote the recovery of hind limb motor function, while *miR-146a-5p* inhibitor could attenuate the recovery function of zhenbao pill. Moreover, *miR-146a-5p* inhibitor could reverse the promoting effect of zhenbao pill on *miR-146a-5p* and the inhibitory effect of zhenbao pill on GPR17 (Figure 7B).

## Discussion

SCI is a complication of spinal fractures, and its development and pathophysiology is a complex process. Neuronal cell apoptosis is the main cause of neurological dysfunction after ASCI [30]. Therefore, how to promote the regeneration of nerve after SCI is the hotspot of current research.

Zhenbao pill is a Mongolian medicine, which can repair damaged nerve cells and scavenge oxygen-free radicals [6]. In this study, animal experiments found that in ASCI rats, zhenbao pill could significantly improve the BBB score, and effectively promote the recovery of hind limb motor function, suggesting that zhenbao pill had a protective effect on spinal cord function after ASCI and promoted the recovery of spinal cord function. However, the present study did not explore which Chinese medicine of zhenbao pill contributed to the protective effect. This part would be explored in the future experiment.

Additionally, the study also found that zhenbao pill could reduce the neurones’ apoptosis after ASCI, suggesting that zhenbao pill had a certain effect on nerve repair, which was consistent with the previous reports. Then, we further studied the mechanism of zhenbao pill on inhibiting neurones’ apoptosis. The results showed that zhenbao pill could up-regulate the level of *miR-146a-5p* in ASCI rats and down-regulate the level of GPR17, which suggested that zhenbao pill might exert its anti-apoptotic effect by regulating *miR-146a-5p* and GPR17 expression.

MiRNA, as a widely existing organism regulatory substance, plays an important role in cell proliferation, differentiation, and cell apoptosis. Studies have shown that many miRNA expressions have changed after ASCI. Strickland et al. [31] showed that 32 miRNAs were significantly down-regulated in the spinal cord tissue of ASCI mice, and three kinds of miRNA were significantly up-regulated. Sung et al. [32] found that *miR-133b* could affect axonal regeneration through the regulation of RhoA protein after SCI, and then play a role in the postinjury repair process. Jee et al. [33] reported that *miR-20a* was highly expressed in SCI mice and could lead to persistent degeneration of motor neurones. Moreover, it was reported that *miR-146a-5p* expression was markedly decreased in spinal cord [17] and could alleviate neuropathic pain in spinal cord [16]. In this study, we had the same findings. We found decreased *miR-146a-5p* expression in ASCI rats, thus we speculated that *miR-146a-5p* might be related to ASCI. GPR17 is thought to be one of the three genes specifically expressed in adult hippocampal neuronal progenitor cells (NPCs), which suggests that it is important in the brain repair process [34]. Franke et al. [35] considered GPR17 as a target for neurorepair. Ceruti et al. [18] reported that GPR17 participated in the regulation of SCI. In the present study, we found that the expression of GPR17 was up-regulated in ASCI rats. Additionally, the study showed that zhenbao pill could up-regulate *miR-146a-5p* and down-regulate GPR17 in SH-SY5Y cells. SH-SY5Y cells were used as a cell model because it had similar characteristics as nerve cells and it was commonly used in spinal cord study [29,36]. Our study found that zhenbao pill could inhibit apoptosis of SH-SY5Y cells. In combination, it suggested that zhenbao pill might exert its anti-apoptotic effect by regulating *miR-146a-5p* and GPR17 expression. Moreover, bioinformatics analysis showed that GPR17 3′-UTR had a binding site with *miR-146a-5p*. The study further demonstrated that GPR17 was a target of *miR-146a-5p*, and could be negatively regulated by *miR-146a-5p*. Interestingly, similar results had been found in cell experiments. In SH-SY5Y cells, zhenbao pill up-regulated the level of *miR-146a-5p* and down-regulated the level of GPR17. And knockdown *miR-146a-5p* could reverse the inhibitory effect of zhenbao pill on GPR17 expression, the anti-apoptotic effect of zhenbao pill and the recovery of zhenbao pill on hind limb motor function in ASCI rats.

However, there are still some shortcomings in the present paper. For example, we did not clear whether the increased expression of GPR17 was causative of the ASCI or simply an epiphenomenon. We would investigate it in the future research.

In summary, zhenbao pill could inhibit neurones’ apoptosis by regulating the expression of *miR-146a-5p*/GPR17, and then promoting the recovery of spinal cord function. This provided a new direction for the treatment of ASCI.

## Abbreviations

ASCI: acute spinal cord injury
BBB: Basso, Beattie, Bresnahan
DLR: double luciferase reporter
GPR17: G-protein-coupled receptor 17
IL-1β: interleukin 1β
iNOS: inducible nitric oxide synthase
LRP: luciferase reporter plasmid
MUT: mutant
NC: negative control
SCI: spinal cord injury
SD: standard deviation
TNF-α: tumor necrosis factor α
WT: wild-type
